# Influence of spurious resonances on the interaction force in dynamic AFM

**DOI:** 10.3762/bjnano.6.42

**Published:** 2015-02-10

**Authors:** Luca Costa, Mario S Rodrigues

**Affiliations:** 1 ESRF, The European Synchrotron, 71 Rue des Martyrs, 38000 Grenoble, France; 2 CFMC/Dep. de Física, Universidade de Lisboa, Campo Grande 1749-016 Lisboa, Portugal

**Keywords:** acoustic excitation, amplitude modulation, atomic force microscopy, fluid borne excitation, interferometric detection, laser-beam detection, spurious resonances

## Abstract

The quantification of the tip–sample interaction in amplitude modulation atomic force microscopy is challenging, especially when measuring in liquid media. Here, we derive formulas for the tip–sample interactions and investigate the effect of spurious resonances on the measured interaction. Highlighting the differences between measuring directly the tip position or the cantilever deflection, and considering both direct and acoustic excitation, we show that the cantilever behavior is insensitive to spurious resonances as long as the measured signal corresponds to the tip position, or if the excitation force is correctly considered. Since the effective excitation force may depend on the presence of such spurious resonances, only the case in which the frequency is kept constant during the measurement is considered. Finally, we show the advantages that result from the use of a calibration method based on the acquisition of approach–retract curves.

## Introduction

Dynamic atomic force microscopy (AFM) was introduced in the late 1980s [1] as the natural evolution of the first atomic force microscopes [2]. Thanks to its flexibility, amplitude modulation AFM (AM-AFM) [3] has become successful and widely employed to characterize surfaces at the nanoscale. It has continuously evolved in terms of achievable lateral resolution and scan speed, producing impressive results both at solid/gas interfaces under ambient conditions [4] and at solid/liquid interfaces [5]. A complete overview is given in [3] and [6]. In AM-AFM, micro-sized cantilevers are conventionally excited at a frequency close to their first eigenmode. The oscillation amplitude of the tip is the feedback signal that is kept constant to obtain the sample morphology during the scan. Obviously, the cantilever excitation plays a central role in AM-AFM. Conventionally, mechanical vibration of the cantilever holder is provided through the excitation of a small piezoelectric element (dither). The setup is widely employed in many commercial and custom-made AFMs and permits measurements both in air and in liquids. Despite the success of this method, the cantilever transfer function presents a forest of spurious peaks particularly when measuring in liquid media. As a consequence, a quantitative estimation of the interaction between the probe and the sample is complicated. Moreover, it has already been observed that the motion of the cantilever base due to the acoustic excitation is not negligible in situations in which the *Q* factor is low – a typical situation when measuring in liquids. The same holds if the cantilever is not excited close to its resonance frequency. Several solutions have been proposed to overcome the presence of spurious peaks in the cantilever transfer function. One possibility is the development of custom-made liquid cells which limit these excitations [7]. Another possibility is the direct excitation of the tip bypassing the conventional piezoelectric excitation. In particular, in the last decade magnetic [8–9], capacitive [10] and photothermal [11–12] actuation schemes have been introduced. The differences between the direct excitation of the tip and the conventional dither excitation have already been studied and reported [13–14]. Consistent efforts have been made to properly quantify the conservative and dissipative interactions when using acoustically excited cantilevers in liquids [15–16]. Here we show that direct excitation is not needed if the position of the cantilever is detected instead of the cantilever bending angle. We report a general study of the dynamics of excited cantilevers and lay-down formulas for deriving the conservative and dissipative interactions considering different types of detection methods. Additionally, this work addresses the consistent advantages that result from the use of approach–retract curves as calibration method [17–19] compared to the standard characterization of the cantilever transfer function.

## Results and Discussion

### Interaction stiffness and damping

In this section we review two general formulas for the interaction stiffness *k*_i_ and damping γ_i_ without using the assumption that the whole system has a specific transfer function, and assuming only that all the forces involved are additive. However, one should note that if we talk of the interaction stiffness, *k*_i_, this contains the implicit assumption that the interaction force, *F*_i_, in the vicinity of the tip oscillation can be expressed approximately as *F*_i_ = *F*_0_ − *k*_i_*x*, with *F*_0_ being a constant and *x* being the tip position.

Consider a point mass which is being acted by two forces. One unknown force given by *F_y_*(*t*) and another known force *F_x_*(*t*) = *A_x_* cos(ω*t*). The two forces add together to give a resultant force 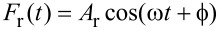, which determines the motion of the mass: 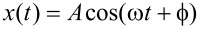, which from Newton’s second law implies *A*_r_ = −*mA*ω^2^. Hence, from basic trigonometric relationships:

[1]
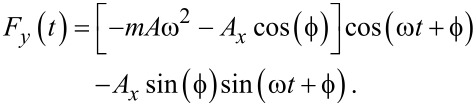


Consider that the force *F_y_*(*t*) has two contributions, a restoring force *F*_rest_ and a damping force *F*_damp_, so that *F_y_*(*t*) = *F*_rest_(*t*) + *F*_damp_(*t*). The restoring force is directly proportional to the position of the moving mass, whereas the damping is directly proportional to its velocity. Let us define *k* as being the proportionality constant between the force and the position and γ the proportionality constant between the damping force and the speed of the mass. Hence,

[2]
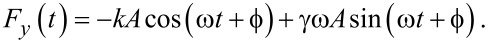


Comparing Equation 2 with Equation 1 gives

[3]
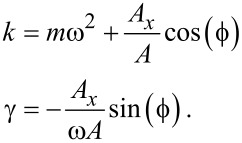


Suppose that the proportionality constant *k* depends on an external parameter *z* so that it can be set *k* = *k*_0_ + *k*_i_(*z*), and in the same way γ = γ_0_ + γ_i_(*z*). In this case

[4]
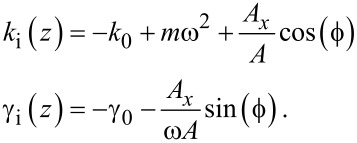


Setting *k*_i_(∞) = 0 and γ_i_ (∞) = 0 yields

[5]
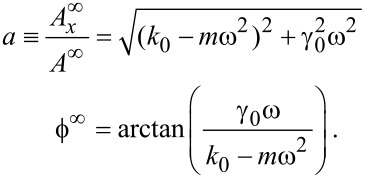


The superscript “infinity” above means that those constants are evaluated far from the surface. In practice, due to the squeeze film effect, they have to be evaluated just before the short range forces occur. We can then write a final relationship:

[6]
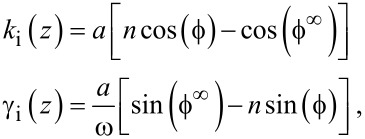


where

[7]
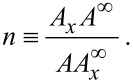


The amplitudes of excitation and tip motion have been normalized to *n* which is *n* = 1 far from the surface. As long as the excitation force remains directly proportional to the excitation signal, it is not necessary to know its actual value, nor how is the cantilever excited. Thus, as far as the quantifying forces is concerned, squeeze film effects or spurious peaks will not affect the quantification of forces. We will come back to this later when considering specifically spurious forces.

If the cantilever spectrum shows a well defined transfer function, then it is straightforward to obtain *a* and 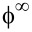 needed to quantify the interaction forces. If the spectrum however is deformed by spurious resonances, then the constants *a* and 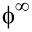 cannot be evaluated from a simple analysis of the spectrum. This does not mean, however, that Equation 6 is incorrect. An important note is that even if the resonance curve is calibrated close to the sample, we assume that *k* is the spring constant of the cantilever, so that a resonance frequency different from the natural frequency is accounted for only through a rescaling of the effective mass and quality factor. Whereas, if *a* and 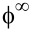 are calibrated, the cantilever spring constant is not fixed to any value.

The reasoning above assumes that the measurement corresponds to the position of the particle to which the forces are applied. Most of the AFMs employ however optical beam deflection schemes [20], providing the measurement of the tip bending angle instead of the tip position. If the cantilever is directly excited so that the base of the cantilever is not displaced [8–12], then Equation 6 is still valid, because in that case the deflection is indeed proportional to the position. If the tip position is not measured and the cantilever is not directly excited, then Equation 6 does not hold, particularly away from the resonance frequency or when the *Q* factor is small.

### Coupling with additional resonances

Consider now that the cantilever is coupled to another oscillator giving rise to one extra peak in the spectrum. The cantilever may be coupled in different ways. Let us consider firstly the case where the coupling is described by

[8]
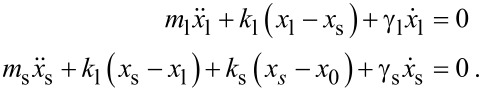


This case corresponds to situations in which additional resonances in the cantilever transfer function are due to the piezoelectric dither in the cantilever holder or due to the cantilever holder itself. The subscript “l” stands for lever and “s” for spurious. Equation 8 is straightforward to solve considering spurious peaks at frequencies close to the cantilever frequency and assuming that nothing else in the system is as soft as the cantilever. If the frequency is comparable to the cantilever resonance frequency, then the ratio *k*_s_/*m*_s_ is also comparable. If the spurious motion is comparable to the cantilever motion, then the situation is such that the term *k*_l_(*x*_s_−*x*_l_) is negligible when compared to the other terms. This implies that the spurious motion is insensitive to motions of the cantilever, i.e., the spurious motion is the same regardless of the cantilever vibrations. Thus, this motion depends only on the excitation signal. As a matter of fact, if the set-up cannot fulfill this requirement, then the cantilever spring constant can not be used for any quantitative evaluation of the interaction, because in this case the cantilever deflection is not directly proportional to the inverse of its spring constant. Therefore the above condition/simplification must, and it does, correspond to the real situation. The above implies Equation 8 can be rewritten as:

[9]
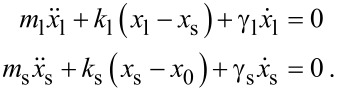


The second equation has a very well-known steady-state solution. Given the amplitude 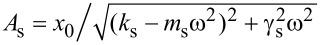 and phase 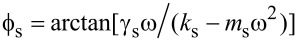, a spurious oscillation will occur. The problem then simplifies and can be summarized in the solution of

[10]
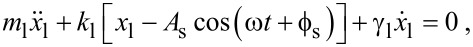


which has the following steady state amplitude and phase:

[11]
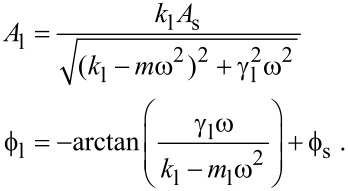


Clearly, at a given frequency, the effect of this spurious resonance is that the excitation signal is amplified (*A*_s_) and a phase lag is introduced. However, if the frequency is kept constant, this amplification factor and phase lag remain constant, hence the quantification of the interaction forces is not affected.

Let us consider now that the liquid motion results in an additional force on the cantilever that does not depend on the cantilever bending, i.e., the fluid is moving at the excitation frequency but it does so, independently of the tip oscillation, as in

[12]
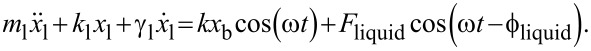


Note that the amplitude of *F*_liquid_ may be a function of the excitation frequency and in particular present resonances, but because we fixed the excitation frequency, this does not matter. If *c*_1_ and *c*_2_ are the proportionality constants between the excitation signal and *kx*_b_ and *F*_liquid_, respectively, then the amplitude and phase of the force acting on the tip are given by:

[13]
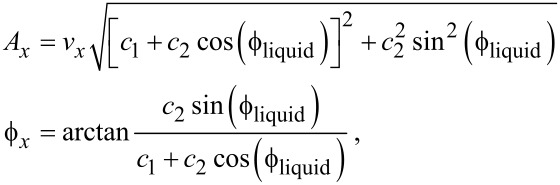


where *v_x_* is the output voltage used to excite the cantilever. If the value inside the square root remains constant then it cancels out when the amplitude is normalized. It is obvious then that such a force will not have any effect in the quantification of the forces if Equation 6 is used. The value of *F*_liquid_ and, hence, of *c*_2_ may however change locally depending on the tip being far away from the sample or at its vicinity or even depending on the sample itself. Such changes will cause variations in both the measured amplitude and phase according to the equations above, hence, explaining changes in amplitude and in phase observed for example in [21]. Therefore, *a*, and 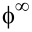 must be evaluated close to the sample surface and assume they remain approximately constant at very short tip–sample distances where the actual interaction takes place.

**Figure 1:**
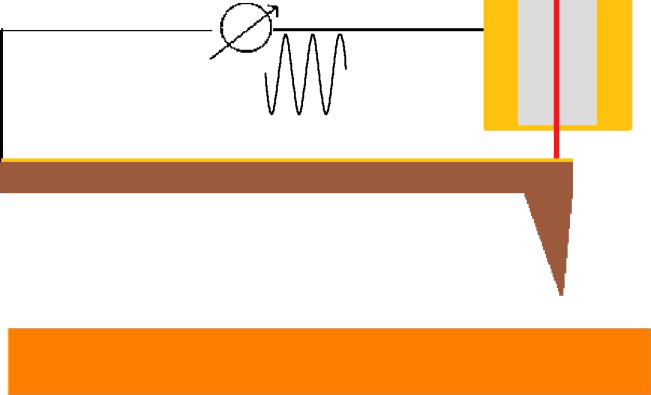
Setup employed for the direct excitation of the tip. A gold coated optical fiber is used to measure the tip position and to apply an electrostatic excitation to the conductive cantilever at a frequency close to resonance.

To experimentally demonstrate the assumptions above we coated an optical fiber with 30 nm of gold at its ends and 300 nm of gold at its edges as illustrated in Figure 1.

**Figure 2:**
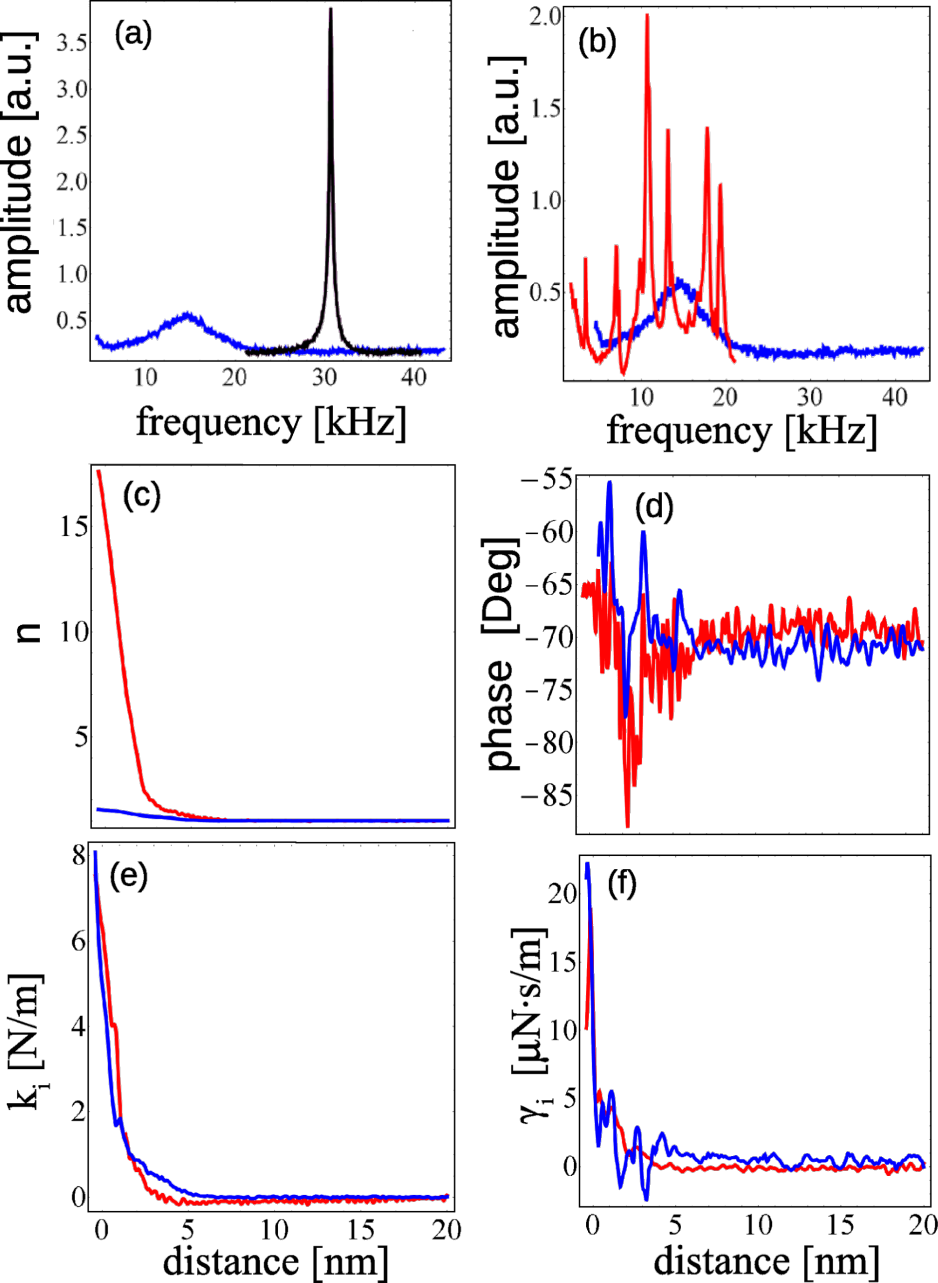
Excitation of the tip in air and in liquid with different actuation methods: a) electrostatic excitation of the cantilever in air (black) and in liquid (blue); b) Amplitude of the tip directly excited (blue) and mechanically excited (red) in liquid; (c and d) normalized excitation and phase, respectively; e) force gradient and f) dissipation measured at the mica/deionized water interface with electrostatic excitation of the tip (blue) and conventional piezoelectric excitation (red).

We applied a harmonic oscillating signal *V*(ω) around a given Δ*V*_0_ which results in a direct electrostatic actuation of the tip. Figure 2a shows the resonance of the excited cantilever in air (black) and in deionized water (blue). The cantilevers have a nominal spring constant of 0.8 N/m. Figure 2b shows the resonance of the cantilever in liquid with direct excitation (blue) and with a conventional piezoelectric excitation (red), showing the presence of spurious resonances.

Approach force curves were acquired in force feedback mode [17–18] at the mica/deionized water interface. The oscillation amplitude of the tip was 0.3 nm. Amplitude and phase were recorded and converted into conservative and dissipative interactions by using Equation 6 and the equality *F* = −∫*k*_i_d*z*. The same measurement was repeated while using conventional piezoelectric excitation instead. Even in the presence of spurious peaks, we converted the amplitude and phase into conservative and dissipative interactions. The force gradient and the dissipation are shown in Figure 2e and Figure 2f. In both cases it is possible to observe the same results in terms of force gradient and dissipation, the main difference between the two excitation methods being only the calibration parameters *a* and 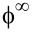, which were in the case of electrostatic excitation 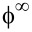 = −0.6 rad and *a* = 2 N/m, and for the conventional acoustic excitation 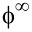 = −0.3 rad and *a* = 0.2 N/m.

### Measurement based on the deflection angle

If the measurement is not directly proportional to the position of the tip but to the cantilever deflection, then the displacement of the base has to be added to the measured signal. Considering Equation 4 and recomputing both 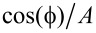 and 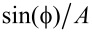, the position of the moving mass, given by its deflection plus the motion of the base, is 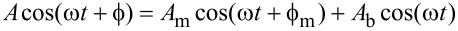, where *A*_m_ is the amplitude of the measured signal and *A*_b_ the oscillation amplitude of the cantilever anchoring point. To simplify, as both *A*_b_ and *A_x_* are proportional to the excitation signal, we can put *A_x_*α = *kA*_b_ where α is zero in case of direct excitation. We can also define a proportionality constant between the measured amplitude and the excitation *nrkA*_m_ = *A_x_* where *n* is again the normalized amplitude and *r* is a constant such that *n* = 1 in the absence of interactions. Equation 4 becomes

[14]
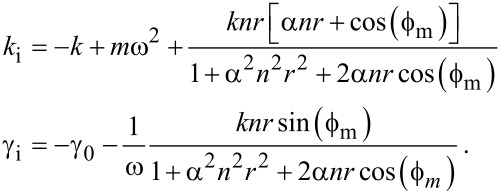


Note that Equation 14 is the same as Equation 4 if one of the following is true:

1. The measured signal corresponds to the position of the moving tip, in which case the terms with α do not appear in the equation.
2. The base of the cantilever does not move (direct excitation), in which case α = 0.
3. It is possible to state that *A*_m_ *>> A*_b_, which implies α ≈ 0.
4. If there is a liquid cell resonance such that *F*_liquid_ *>> kA*_b_, in which case the cantilever behaves as being directly excited.

If none of the above is true, then there is one particular noteworthy case that corresponds to an excitation force solely due to the cantilever base displacement. Note that this implies the absence of spurious forces. In that case α = 1 and Equation 14 simplifies to

[15]
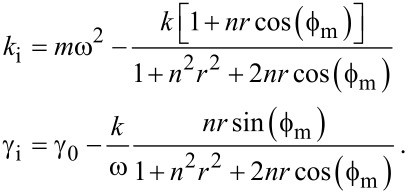


Far from the sample surface, Equation 15 must equal zero. We can solve Equation 15 to find the constants 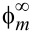 and *r*:

[16]
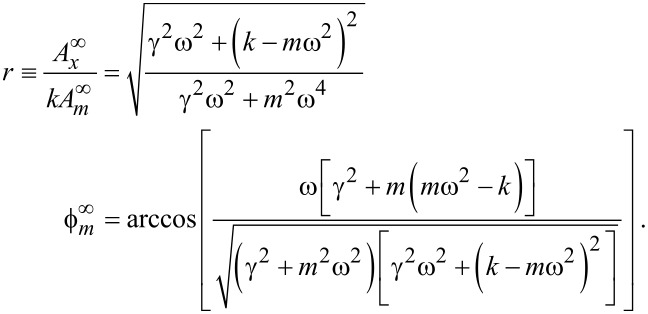


The first terms in Equation 15 can be computed from the information far from the sample:

[17]
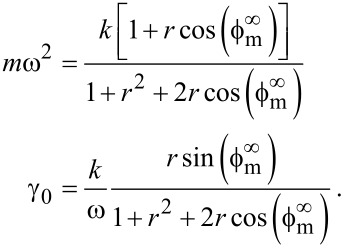


This highlights the fact that if the cantilever spring constant *k* is known, then the evaluation of either γ and *m*, or *r* and 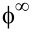 is needed to quantify the interaction. The first couple of constants can be found from a resonance curve if it is well-defined in the spectrum. The second couple can be found from a calibration curve obtained from a known force or by using for example an approach curve and the fit of *F* = −∫*k*_i_d*z* as in [17–18]. In air it is straightforward to calibrate these constants by using for instance an electrostatic force.

**Figure 3:**
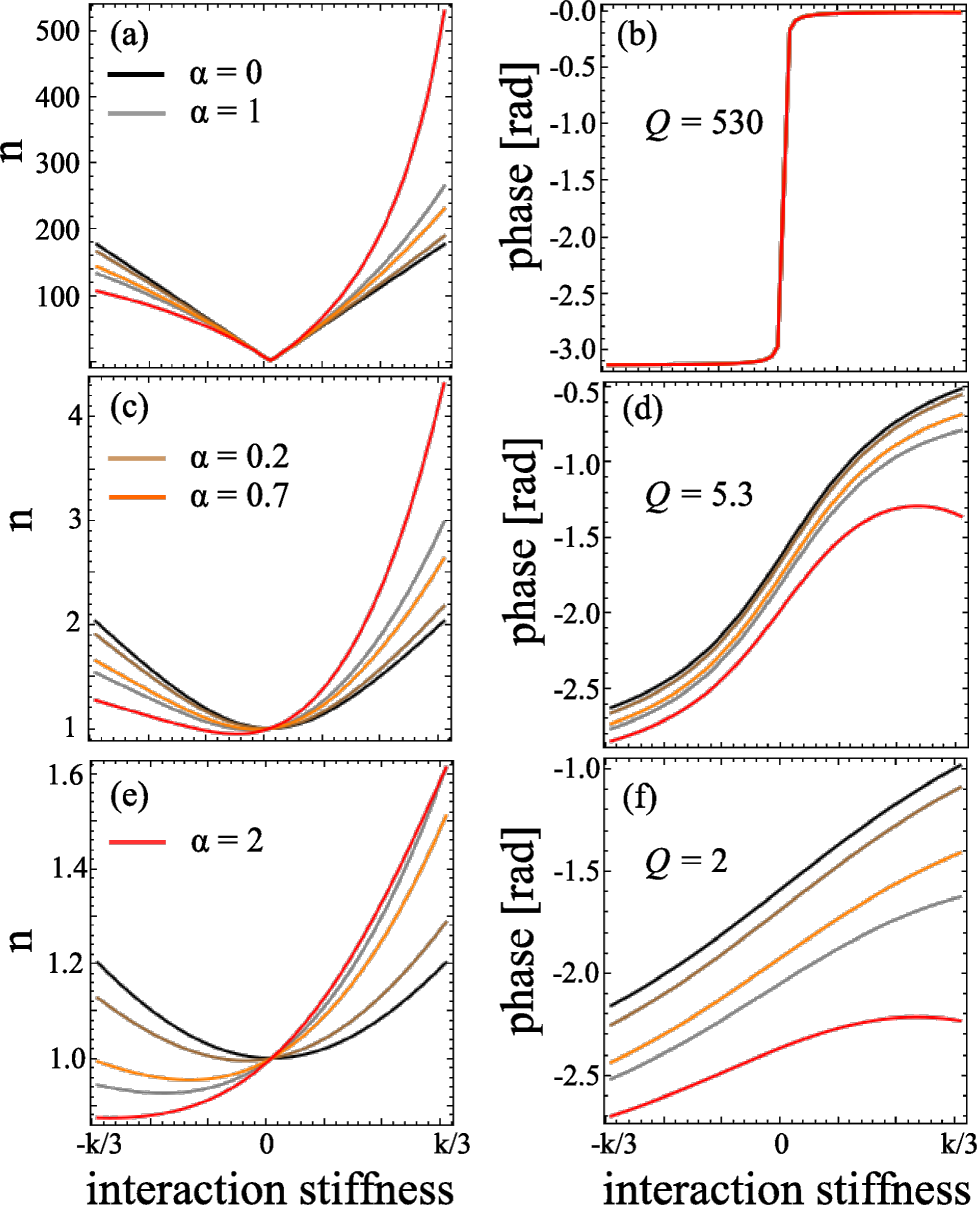
Equation 14 simulating amplitude (left) and phase (right) for three different quality factors from high (top) to low (bottom). Each plot contains five curves, each corresponding to a different α. If α = 0 (black) then the solution is equal to that given by Equation 6, for α = 1 (gray) the solution is equal to that given by Equation 15. For 0 < α < 1, the solution lies somewhere between that given by Equation 6 and Equation 15. The red curve shows the solution for α = 2.

Finally, some considerations regarding the case in which α ≠ 1. This case depicts piezoelectric actuation resulting in a non-negligible liquid-borne excitation. First note that this situation poses no difficulty in the case in which the tip position is measured. It is more complicated in the case of beam deflection because Equation 14 is more difficult to analyze. The values of *A_x_* and *A*_b_, or their ratios, are not acquired from a conventional analysis of the transfer function nor from fitting the Brownian motion of the cantilever. One could perhaps use the equality 
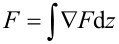
 to calibrate Equation 14. However, it turns out that for 0 < α < 1 the solution lies somewhere between those given by Equation 6 and Equation 17 (see Figure 3). As such, either Equation 6 or Equation 17 can be used to effectively calibrate the required constants. When α *>>* 1, the situation is more complicated. Fortunately, this will happen only in very particular cases. Going back to Equation 13 one concludes

[18]
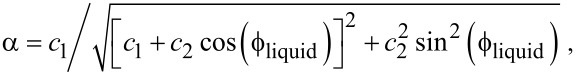


which means α *>>*1 only when *c*_1_ ≈ *c*_2_ and 
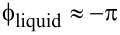
.

To further prove the statements in the last paragraph, and because one usually does not measure α, we have considered a force gradient going from −*k*/3 up to *k*/3 for different α by using Equation 14, Equation 6 (α = 0) and Equation 17 (α = 1). The latter was done considering three cantilevers all with the same resonance frequency but with rather different *Q* factors from high to low. In Figure 3 we show what would be the amplitude and phase measured for five different values of α (represented with different colors) and for three different *Q* factors. For high *Q* factors and small interactions all expressions lead to about the same values (Figure 3), meaning that in such cases it is not necessary to worry about the constant α. However, for lower values of *Q* the solutions give quite different results. Figure 3 illustrates the rather large errors that can be made if the excitation force is not well taken into account showing the advantages of measuring position rather than deflection.

**Figure 4:**
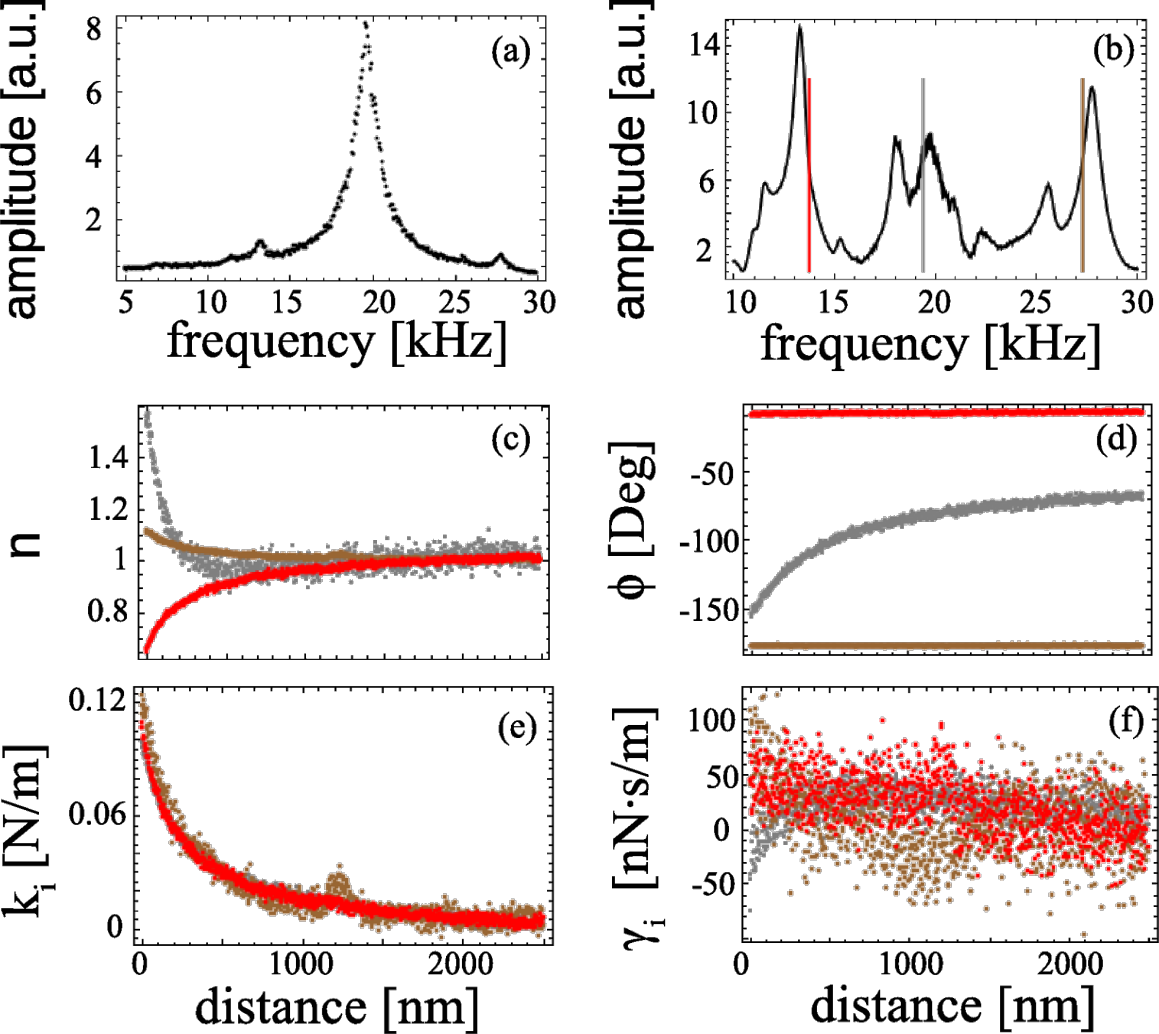
Characterization of a tip–sample electrostatic interaction at resonance (gray) and off resonance (red and brown). a) Cantilever Brownian motion; b) spectrum of the excited cantilever; c) normalized amplitude at three selected frequencies indicated in the spectrum; d) measured phase (offset so that at infinity it gives 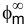); e) and f) force gradient and dissipation measured at the three selected frequencies.

To experimentally illustrate the situation described above, we have prepared a cantilever holder that excites the cantilever by a combination of rotation and translation [22]. In this case *A_x_* ≠ *kA*_b_. Furthermore, this particular holder shows many spurious resonances. We applied an electrical potential difference between a conductive cantilever and a conductive sample which results in an electrostatic interaction. Three approach curves were taken at selected arbitrary frequencies (Figure 4b) and Equation 17 was used to account for the interaction. All the measurements provide the same force gradient (Figure 4e) regardless of the presence of spurious resonances (Figure 4a), whereas the dissipation remains constant and approximately equal to zero. The force was used to obtain all unknown constants.

Interestingly, the values of *r* and 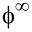 that result from the calibration are those that would be expected if the spurious peaks were not present. Notice in Figure 4d that the phase is close to 0, −90 and −180°, when the cantilever is, respectively, excited below, close to, and above the resonance frequency. To ease the task of calibrating the cantilever, *r* and 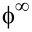 were initially guessed from Equation 16, for which the constants were obtained from the analysis of the tip Brownian motion shown in Figure 4a. The resulting r^*^ and 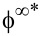 are listed in Table 1 together with *r* and 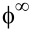 values obtained from the analysis of the Brownian motion. This result shows that the change in amplitude and phase due to the interaction force were about the same, regardless of the presence of spurious peaks.

**Table 1:** Calibration based on matching the integral of the force gradient to the force (r^*^, 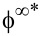), and using directly the constants determined from fitting the Brownian motion of the tip (*r*, 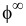).

*f_x_*	13734 Hz	19393 Hz	27315 Hz
*r*	1.05 N/m	0.059 N/m	0.48
r^*^	1.05	0.056	0.4
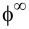	−8°	−66°	−178°
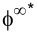	−8°	−66°	−177°

## Conclusion

We have introduced a methodology to directly derive the conservative and dissipative interactions between the AFM probe and the sample in dynamic AFM experiments when small oscillation amplitudes of the tip are used, and for different tip excitation and detection schemes. We considered both direct detection of the tip position, for example with Fabry–Perot interferometers, and classic optical beam deflection scheme, showing that the first method allows a much easier calculation of the interactions. We have proposed a calibration method of the cantilever response that is not based on the measurement of its transfer function, but it is based on the acquisition of the tip position/deflection, amplitude and phase during approach curves and consequently the assumption of the equality *F* = −∫*k_i_*d*z*. The method has been employed to compare the interaction force at the mica/deionized water interface measured with a conventional piezoelectric dither excitation and direct electrostatic excitation. The different setups provide the same evaluation of the force gradient although the calibration parameters are different. Finally, we have highlighted the changes that have to be introduced into the formulas for optical beam deflection based AFMs. With the described procedure, it is possible to ignore the effects of spurious resonances even when using optical beam deflection-based AFMs.
